# The Impact of Image Acquisition Parameters and ComBat Harmonization on the Predictive Performance of Radiomics: A Renal Cell Carcinoma Model

**DOI:** 10.3390/app12199824

**Published:** 2022-09-29

**Authors:** Abdalla Ibrahim, Lin Lu, Hao Yang, Oguz Akin, Lawrence H. Schwartz, Binsheng Zhao

**Affiliations:** 1 Department of Radiology, Columbia University Irving Medical Center, New York, NY 10032, USA; 2 Department of Radiology, Memorial Sloan Kettering Cancer Center, New York, NY 10065, USA

**Keywords:** handcrafted radiomics, harmonization, ccRCC

## Abstract

Radiomics, one of the potential methods for developing clinical biomarker, is one of the exponentially growing research fields. In addition to its potential, several limitations have been identified in this field, and most importantly the effects of variations in imaging parameters on radiomic features (RFs). In this study, we investigate the potential of RFs to predict overall survival in patients with clear cell renal cell carcinoma, as well as the impact of ComBat harmonization on the performance of RF models. We assessed the robustness of the results by performing the analyses a thousand times. Publicly available CT scans of 179 patients were retrospectively collected and analyzed. The scans were acquired using different imaging vendors and parameters in different medical centers. The performance was calculated by averaging the metrics over all runs. On average, the clinical model significantly outperformed the radiomic models. The use of ComBat harmonization, on average, did not significantly improve the performance of radiomic models. Hence, the variability in image acquisition and reconstruction parameters significantly affect the performance of radiomic models. The development of radiomic specific harmonization techniques remain a necessity for the advancement of the field.

## 1. Introduction

With radiomics, quantitative medical image analysis can be performed by extracting quantitative features hypothesized to decode biologic information not even detectable by the human eye [1,2]. The field has rapidly expanded, with large numbers of published studies regarding the potential of radiomics for clinical applications. Determining lesions’ malignant potential [3–5], assessing response to therapy [6–8], and predicting overall survival [9,10] are some of the clinical endpoints investigated.

Despite the many possibilities with radiomics, there are a number of limitations that have hindered the translation of radiomic signatures (models) into clinical practice [11,12]. As is the case of almost all other biomarkers [13], radiomic features (RFs) must be repeatable and reproducible to be used in clinical settings [14,15]. One of the limitations of real-world data is that CT scanners from different vendors, different models of CT scanners from the same vendor, and the imaging center implementation of these CT scanners result in many variations of image acquisition parameters. Previous studies have reported that the reproducibility of RFs can be significantly affected by the variations in image acquisition parameters including scan acquisition and reconstruction settings [15–19]. Therefore, it is ideal to evaluate and potentially “correct” these settings in order to provide the most accurate radiomics analyses. Two potential solutions for these problems include (1) the selection and exclusive use of robust RFs by studying the results of reproducibility analyses and (2) the reduction of the variations in RF values not attributed to biologic differences [14] by either pre-processing, for example image resampling, and/or post-processing, for example with ComBat harmonization techniques [20].

A number of studies have investigated the effects of different preprocessing methods, such as image resampling [16,21,22], kernel normalization [23–25], and image discretization [26–28]. These studies reported inconsistent performance depending on the data analyzed. ComBat harmonization, a method originally developed for removing batch effects from gene expression arrays [29], has been investigated as a potential harmonization method in radiomics analyses. ComBat is a function that is based on empirical Bayes and attempts to remove the effects attributed to machinery differences while preserving biologic information. This is done by pooling the gene expressions and providing ComBat formula with the biologic covariates that are known to affect the expression of genes being harmonized [29]. However, there are several limitations to fulfilling the assumptions of ComBat in radiomics analyses. First, radiomic features are differently affected by the variations in imaging parameters as previously reported. Therefore, pooling all RFs in a single ComBat harmonization run might affect the performance of ComBat. Second, the aim of radiomic analyses is to investigate the correlations with biologic information. As such, it is not possible to provide the correct biologic covariates to the ComBat formula, risking the loss of biologic information in the process. Lastly, ComBat is a data-driven harmonization method, i.e., the estimates of batch effects can vary significantly with the variations in the data being harmonized. In this manner, every time new data point are added, the harmonization and modeling process have to be repeated [30], which makes the application of ComBat not suitable for clinical practice without specific adjustments of ComBat for RFs. Hence, we are performing this experiment to evaluate these hypotheses.

Clear cell renal cell carcinoma (ccRCC) is the most common subtype of renal adenocarcinoma, the most common type of renal malignancies which account for around 90% of kidney cancers [31]. ccRCC has variable outcomes for individual patients, and consequently, the predictability of overall survival in ccRCC patients remains challenging clinically [32]. RFs could be of potential utility for predicting overall survival in these patients. Previous studies reported promising results [30,33–35]. However, the translation to clinical applications remains extremely challenging due to the above-mentioned limitations. For example, a previous study utilized the same dataset to assess to which degree do the variations in imaging parameters affect the performance of radiomic signatures [30]. The study concluded that the generalizability of radiomic signatures can be negatively affected by multiple factors, such as the differences in slice thickness and tumor size, however, more insight is needed.

In this study, we aim to assess the potential of RFs to predict overall survival in ccRCC patients, using real-world, heterogeneously acquired CT scans and in addition, we also assess the impact of different approaches of ComBat harmonization on the performance of RFs to predict overall survival.

## 2. Materials and Methods

### 2.1. Imaging Data

The analyzed data in this study are publicly available on The Cancer Imaging Archive (TCIA) [36]: (https://wiki.cancerimagingarchive.net/display/Public/TCGA-KIRC, accessed on 3 April 2022) [37]. We utilize 267 ccRCC patients collected from multiple medical centers nationwide. Of these, 179 CT scans were included in the analysis based on the availability of both the imaging and survival data. The imaging vendors and parameters for this data set are summarized in Table 1.

Additionally, the data contained scans that were acquired in different contrast-enhancement imaging phases (70 arterial scans and 109 portal venous scans). Therefore, the data was analyzed in three ways and three datasets were created accordingly: (i) All of the data—Dataset 1; (ii) Arterial scans only—Dataset 2; and (iii) Portal venous scans only—Dataset 3.

### 2.2. Tumor Segmentation and Feature Extraction

An abdominal radiologist who was blinded to the patients’ clinical information segmented the renal lesions on each scan. A MATLAB-based (MathWorks, Natick, Massachusetts) application was used to visualize and segment the tumors on CT scan. The application is based on a semi-automated algorithm, which combines the region-based active contours and a level set approach, in a slice-by-slice manner. The images were then isotropically resampled to 0.5 × 0.5 × 0.5 mm^3^. A total of 1160 RFs were extracted from each segmented tumor via the Columbia Image Feature Extractor (CIFE) [30].

### 2.3. ComBat Harmonization

ComBat method is an empirical Bayes-based method that is used to estimate the effects of different batches on the quantitative readings. For RFs, ComBat formula assumes that a feature value can be approximated by the equation:

(1) Yij=α+βXij+γi+δiεij

where α is the average value for HRF Yij for ROI j on scanner i;X is a design matrix of the biologic covariates that are known to affect the value of HRFs; β is the vector of regression coefficients corresponding to each biologic covariate; γi is the additive effect of scanner i on HRFs, δi is the multiplicative scanner effect, and εij is an error term, presupposed to be normally distributed with zero mean. Following the estimation of effects, ComBat performs feature harmonization as follows:

(2) $$YijComBat\;=(\left(Yij-\alpha^{\wedge }-\beta^{\wedge }Xij-\gamma i^{*}\right))/(\delta i^{*})+\alpha +\beta^{\wedge }Xij$$

where $\alpha^{\wedge }$ and $\beta^{\wedge }$ are estimators of the parameters α and β, respectively; and $\gamma i^{*}$ and $\delta i^{*}$ are the empirical Bayes estimates for the parameters γi and δi, respectively [38]. ComBat harmonization was applied in this study using the slice thickness and pixel spacing values as the batch. For the slice thickness, the scans were divided into thin slices (≤3 mm) and thick slices (>3 mm). For the pixel spacing, the scans were divided into three groups: between 0.5 and 0.7 mm, between 0.71 and 0.80 mm, and between 0.81 and 0.98 mm. The scans were acquired using similar convolution kernels in the majority of the scans, making the number of observations for the minority kernels not sufficient for ComBat application. The same applies when the medical center where the scans were acquired is considered as the batch.

### 2.4. Analysis Strategy and Pipeline

**Figure 1.**
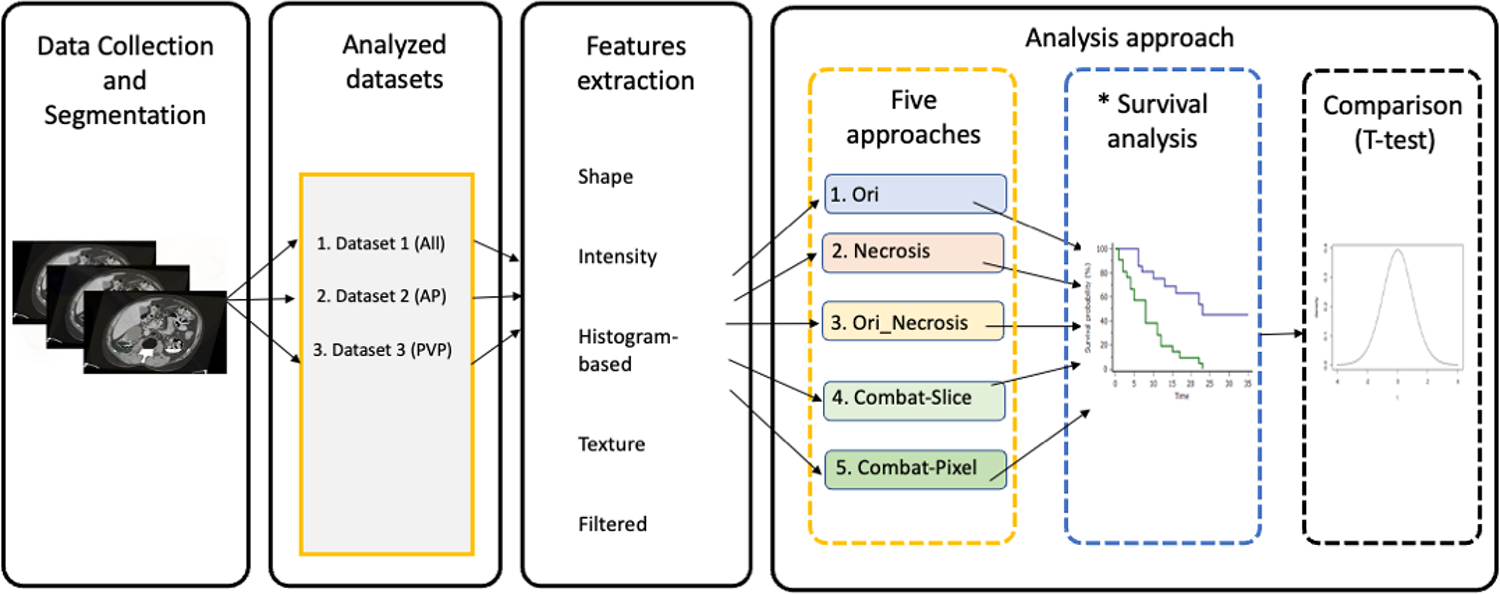
Study workflow. * The survival analysis is performed 1000 analyses per approach.

The workflow consisted of four major steps: the collection and curation of imaging dataset; the division of the data into three datasets as described in Section 2.1; the extraction of RFs; and the statistical analysis. Figure 1 describes the workflow of the study. Fifteen different experiments were performed, and the performance of the different approaches is compared accordingly.

Each dataset was analyzed using five different approaches. In the first approach, RFs extracted from the CT scans were directly used to assess overall survival (Ori). In the second approach, the clinical variable “percentage of tumor tissue necrosis” was used to assess overall survival (Necrosis) [39]. In the third approach, the percentage of tumor tissue necrosis and RFs were combined to assess overall survival (Ori_Necrosis). In the fourth approach, RFs were harmonized using ComBat harmonization, with the slice thickness being the batch, in order to assess overall survival. In the fifth approach, pixel spacing was used as the batch to harmonize RFs with ComBat, and the harmonized RFs were used to predict overall survival. To avoid reporting significant results by chance, the analysis was repeated 1000 times in each of the approaches. In each repeat, the data are split randomly into 70% training and 30% validation sets. Feature selection and cox regression are performed in each repeat. The concordance index (C-index) was used to evaluate the performance of developed cox regression models [40]. The C-index of each of the final developed models on the validation set is kept in each repeat, and an average is calculated on the indices saved per approach. By performing the analysis 1000 times, we investigate whether the selected radiomic features and calculated performances are robust.

The set of features to be included in the modeling was contingent upon which of the five approaches (explained above in strategy) was utilized. In each of the repeats, following the division of data into training and validation sets, highly correlated features (identified using Spearman’s correlation [41] with a cut-off of R > 0.90) were removed. If two RFs were found to be highly correlated, the RF that was removed was the one with higher average correlation with the other RFs. The remaining RFs were then used to build a cox regression model [42]. Backward feature selection [43] was applied on the generated cox model to select the important features. The selected features were then used to build the final cox regression model. Due to the small dataset size, if the number of the selected features was higher than 5, the 5 features with the most contributions to the model were used to build the final model. The model performance was then validated on the validation set using the C-index. Student *t*-test [44] was used to assess the difference on average between the different approaches used. All statistical were performed using R [45] language on RStudio (Version 2022.2.1.461, RStudio Inc., Boston, MA, USA) [46].

## 3. Results

### 3.1. Patient Characteristics

In this study, 179 out of 267 patients were included in the final analysis based on the availability of pre-surgical CT scans and survival data. The patients included had a median age of 59 years, 119 (66.5%) were male, and 109 (60.9%) were diagnosed at Stages I and II, with an average (±standard deviation (SD)) overall survival of 41 ± 24.7 months. The patients included received different treatment regimens, including immunotherapy, targeted molecular therapy, and chemotherapy.

### 3.2. Performance of Original RFs and Percentage of Tumor Tissue Necrosis

**Figure 2.**
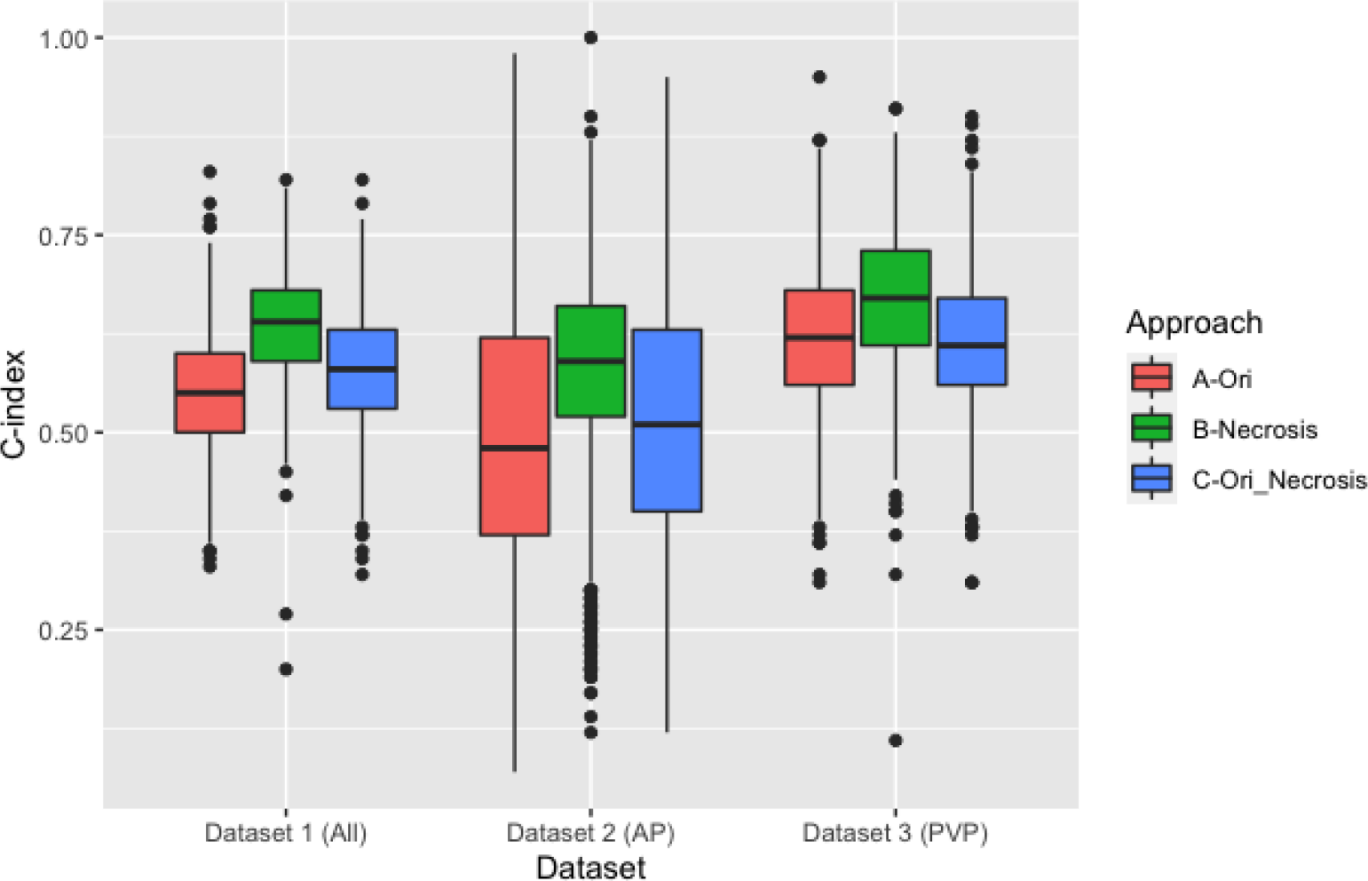
Boxplot comparison of the C-indices of the clinical and original RF models.

The results of the different analysis approaches varied slightly according to the data being analyzed and followed similar patterns (Figure 2). For the first approach, when Dataset 1 was analyzed, the performance of RFs (C-index) across the 1000 repeats ranged between 0.33 and 0.83, with an average (±SD) of 0.55 ± 0.08. The RF “Intensity minimum” was selected in 91.8% of the times, followed by intensity skewness (77.3% of the times), intensity peak position (70.6%), and intensity kurtosis (64.8%). The remaining features were selected in less than 50% of the times.

In Dataset 2, the c index values across the 1000 repeats ranged between 0.07 and 0.98, with an average (±SD) of 0.50 ± 0.18. None of the features was selected more than 50% of the times. Intensity minimum and intensity kurtosis were the most selected features (49.5 % and 46%, respectively).

In Dataset 3, the C-index values ranged between 0.31 and 0.95, with an average (±SD) of 0.62 ± 0.09. None of the features was selected more than 50% of the times. Intensity minimum and intensity kurtosis were the most selected features (49.5 % and 46%, respectively). Intensity minimum was the most selected RF (79.2%) followed by intensity skewness (61.4%) and intensity peak position (52.2%).

For the second approach, we assessed the ability of the clinical variables “percentage of tumor tissue necrosis” to stratify the patients in long and short survivors. The C-index values ranged (average ± SD) between 0.20 and 0.82 (0.64 ± 0.07), 0.12 and 1.00 (0.58 ± 0.12), and 0.11 and 0.91 (0.67 ± 0.09) in Dataset 1, Dataset 2, and Dataset 3, respectively.

For the third approach, combining the percentage of tumor tissue necrosis with the RFs resulted in C-indices with an average (±SD) of 0.58 ± 0.07, 0.52 ± 0.16, 0.61 ± 0.09 in Dataset 1, Dataset 2 and Dataset 3 respectively.

*T*-tests showed that on average, the percentage of tumor tissue necrosis significantly (*p*-value < 0.05) outperformed the RFs-based models as well as the combination of RFs and the percentage of tumor tissue necrosis across all the three datasets.

### 3.3. Impact of ComBat Harmonization

**Figure 3.**
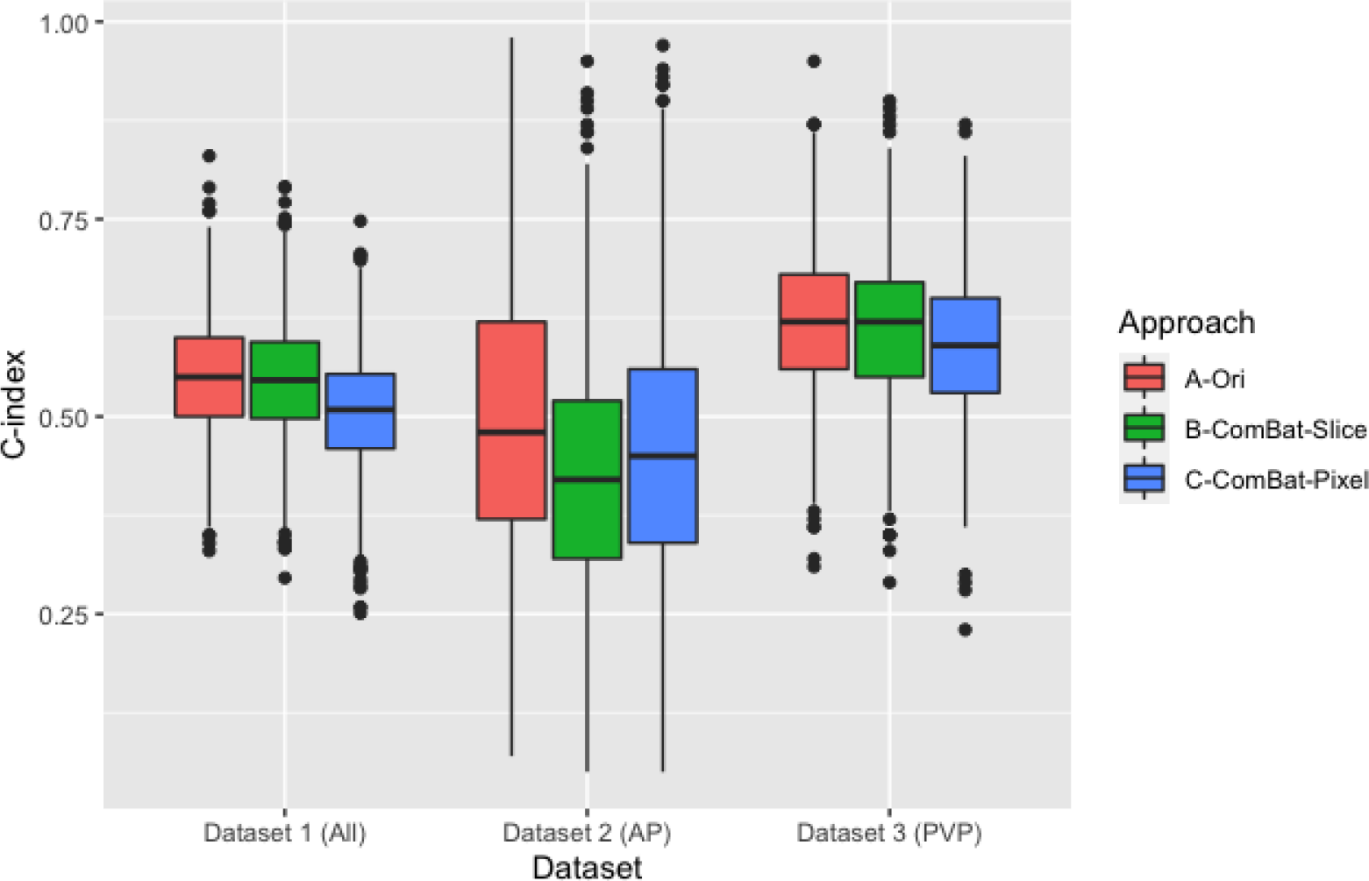
Boxplot comparison of the C-indices before and after ComBat harmonization.

For the fourth approach, the slice thickness was used as the batch (Figure 3), the C-index values ranged (average ± SD) between 0.30 and 0.79 (0.55 ± 0.08) in Dataset 1, with intensity maximum being the most selected feature (94.8% of the times), followed by intensity peak position (84%) intensity minimum (79.2%), intensity skewness (59%), and intensity uniformity (51.4%).

In Dataset 2, the c index values range (average ± SD) was 0.05–0.95 (0.43 ± 0.16), with none of the features being selected in more than half of the times. Intensity minimum was the most selected feature (43.7%) followed by intensity kurtosis (35.6%). In Dataset 3, the c index values ranged between 0.29 and 0.90 with an average (± SD) of 0.61 ± 0.09. Intensity minimum was the most selected feature in this scenario (76%) followed by intensity peak position (68.2%). The remaining RFs were selected in less than 50% of the times.

Using the pixel spacing the batch (the fifth approach) resulted in C-index values ranges (mean ± standard deviation) of 0.25–0.75 (0.51 ± 0.08), 0.05–0.97 (0.47 ± 0.17), and 0.23–0.87(0.59 ± 0.09) in Dataset 1, Dataset 2, and Dataset 3, respectively.

On average, the models based on the original RFs values before ComBat harmonization significantly (*p*-value < 0.05) outperformed the models based on harmonized RFs across all datasets, except for the models based on RFs harmonized based on the slice thickness in Dataset 1.

## 4. Discussion

In this study, we aimed to investigate (i) the potential of RFs to predict the overall survival in ccRCC, without adjusting for the differences in image acquisition and reconstruction parameters, using thorough statistical analyses; and (ii) the impact of ComBat harmonization on the performance of RFs. We found that on average, radiomic signatures had an above chance level (C-index > 0.5) for the prediction of overall survival in ccRCC patients. Since in machine learning different data splits could result in significant variations in the performance of the model developed, we ran the analyses with thousand different splits and assessed the performance of radiomic signatures. It was observed that the chances of obtaining a C-index of 0.70 or higher across the data and its subsets was less than 5%, which falls within chance level. Moreover, different RFs were selected with different splits of the data. However, three intensity features were selected in at least 70% of the times (Supplementary Table S1). These RFs could be considered correlated with overall survival in ccRCC patients. Nonetheless, definitive conclusions can only be reached following the collection of more datapoints, as well as the availability of reproducibility analysis to assess the robustness of these RFs to the variations in imaging parameters observed across the data being analyzed [14].

Furthermore, we compared the performance of radiomic signatures to that of the percentage of tumor tissue necrosis, since the latter is a known clinical predictor [39,47]. Our results showed that on average, predictions based on the percentage of tumor tissue necrosis alone significantly outperformed those based on the different radiomic signatures. Nonetheless, the most frequently selected RFs (e.g., “intensity minimum”, “intensity skewness”, as shown in the supplement Table S1) were CT attenuation-related features, which more or less reflected some information about tumor tissue necrosis. We noticed that none of the predefined RFs was highly correlated to the percentage of tumor tissue necrosis. The findings that the clinical model only—on average—performed better, suggests that the RFs likely resulted in added noise to the survival model. Nevertheless, the comparison is not representative of the full potential of RFs, since in some instances the performance of the combination was higher, and the limitations of reproducibility have to be addressed before a final conclusion could be reached.

The data analyzed in this study included scans that were acquired with different hardware and different imaging parameters, such as convolution kernel, slice thickness, and pixel spacing. Variations in these parameters have been previously reported to affect the reproducibility of RFs. A number of studies investigated the effects of variations in convolution kernels on the reproducibility of RFs. All of these studies concluded that the variations in convolution kernel significantly affects the reproducibility of RFs [17,23–25]. Similarly, the effects of variations in slice thickness [23,48–50], pixel spacing [16,50], and effective mAs [51] were reported to have significant effects on the reproducibility of RFs. Since the data analyzed in this study included data acquired with different imaging protocols, it is expected that the reproducibility of the majority of RFs is affected significantly, which could explain the wide range of C-indices obtained across the thousand repeats in each approach. It could also explain the selection of intensity based RFs across all the investigated approaches, since these RFs are expected to be the least affected by the variations in our data. In addition, more sensitive RFs that decode texture were not frequently selected, which is in line with the expectations based on the literature.

We assessed the impact of ComBat harmonization on the performance of RFs using two approaches; one using the slice thickness, and the second using the pixel spacing to group the scans. We have not provided any biologic covariates to the calculations of ComBat estimates, since the aim of radiomics studies is to investigate clinical correlations. We observed on average a decrease in the performance of RFs following ComBat harmonization in both of the approaches. The probability of obtaining a C-index of 0.70 or higher was also within chance level (<5%). When slice thickness was the batch used for ComBat harmonization, three intensity features were selected in 70% or more of the times. Two of these RFs were selected before and after harmonization. On the other hand, when pixel spacing was used as the batch, four intensity features were selected more than 70% of the times, one of which was selected before and after harmonization. In addition, *t*-test analyses showed that the performance of RFs before harmonization was significantly higher. This could be justified by a number of theories. First, the assumption of similar batch effects on the RFs is not met, which could affect the estimation of batch effects. Another possibility is that ComBat harmonization removed the biologic signals in the harmonization process. Regardless of the reason, there was no added value of performing ComBat harmonization in the reported manner in this dataset. This signifies the need for radiomics-specific harmonization methods.

While we designed statistically thorough experiments, a number of limitations are to be acknowledged. First, there was a lack of data to perform reproducibility analyses and preselect reproducible RFs, as well as the lack of a more homogenous dataset to compare the performances. Second, the relatively low number of datapoints analyzed, as well as the different treatment regimens used could adversely affect the performance of the signatures developed, especially on the arterial subset of the data. More datapoints are needed to reach more concrete conclusions. Third, the data were collected from different centers, which could introduce more variations due to the patients’ characteristics and center protocols. The small numbers of patients per center did not allow for adjusting for these potential variations. In addition, given that the scans were resampled before feature extraction, the impact of ComBat harmonization could be impacted. Lastly, other factors that are known to affect the concordance of RFs, such as intra- and inter-observer variabilities need to be assessed in future studies.

## 5. Conclusions

In conclusion, the variations in the imaging parameters in the data under analysis significantly affects the performance of developed radiomic signatures. The application of ComBat harmonization in our data did not improve the performance of radiomic signatures, however reproducibility analyses may enhance the performance of ComBat by identifying specific RFs to utilize. Radiomics-specific harmonization techniques currently remain one of the major needs and challenges in the field of radiomics.

## Supplementary Material

Table S1

## Figures and Tables

**Table 1.** Description of the data included.

Vendor	Number of Scans	Convolution Kernels	Slice Thickness (mm)	Pixel Spacing (mm^2^)

GE	154	Standard, Soft	1.25–7.5	0.7 × 0.7–0.78 × 0.78
Philips	3	A, B	5	0.74 × 0.74–0.86 × 0.86
Siemens	22	B30f, B30s, B31f, B31s	3, 5	0.54 × 0.54–0.98 × 0.98

## Data Availability

The data is publicly available on TCIA.org: (https://wiki.cancerimagingarchive.net/display/Public/TCGA-KIRC, accessed on 3 April 2022).
