# Supplementary material for: The Impact of Image Acquisition Parameters and ComBat Harmonization on the Predictive Performance of Radiomics: A Renal Cell Carcinoma Model
Source: Appl Sci (Basel). Author manuscript; Available in PMC 2023 Apr 21. (PMC10121203; doi:10.3390/app12199824)
Supplement: Table S1 [file NIHMS1887786-supplement-Table_S1.pdf]

Table S1. Most selected radiomic features in the different approaches.

Dataset 1 (All)		
Ori*	ComBat-Slice	ComBat- Pixel
Intensity Minimum	***Intensity Maximum***	***Intensity Kurtosis***
Intensity Skewness	***Intensity PeakPosition***	***Intensity Uniformity***
Intensity PeakPosition	***Intensity Minimum***	***Intensity Skewness***
Intensity Kurtosis	***Intensity Skewness***	***Intensity PeakPosition***
Intensity Skewness	***Intensity Uniformity***	***Intensity Maximum***
Dataset 2 (AP)		
Ori*	ComBat-Slice*	ComBat- Pixel*
Intensity Minimum	Intensity Minimum	Intensity Maximum
Intensity Kurtosis	Intensity Kurtosis	Intensity Minimum
Intensity Maximum	Intensity Maximum	Intensity Kurtosis
Intensity Skewness	Intensity Skewness	Intensity Skewness
Intensity Maximum	Intensity Maximum	Intensity Range
Dataset 3 (PVP)		
Ori*	ComBat-Slice*	ComBat- Pixel
Intensity Minimum	***Intensity Minimum***	***Intensity Minimum***
Intensity Skewness	***Intensity PeakPosition***	***Intensity Maximum***
Intensity Skewness	Intensity Uniformity	***Intensity Range***
Intensity PeakPosition	Intensity Skewness	***Intensity Skewness***
Intensity Maximum	Intensity Maximum	***Intensity Kurtosis***

* Italic bolded features were selected in more than 50% of the runs.
